# Supplementary material for: Molecular Basis of Sperm Methylome Response to Aging and Stress
Source: Biology (Basel). 2026 Mar 21;15(6):504. doi: 10.3390/biology15060504 (PMC13024700; doi:10.3390/biology15060504)
Supplement: Supplementary file 1 [file biology-15-00504-s001.zip › biology-4187235-supplementary.pdf]

Supporting Tables

Table S1. Sperm RRBS datasets used in this study to identify VMRs and DMRs.

Animal model	Analyzed factor	N/ group	Age of animals, PND	Original study	Methylation regions analyzed	Source of original data
Mouse	Age	8	56 vs 154	[41]	VMRs in young and mature mice; age-dependent DMRs	[44]
Mouse	BTB disruption via Sertoli-specific Rictor KO	4	154	[41]	DMRs induced by BTB disruption	[44]
Mouse	Adult exposure to 0.69 mg/kg/day $CdCl_2$ for 2 spermatogenesis cycles	15	119	[9]	DMRs induced by $CdCl_2$	GEO accession number: GSE158455
Mouse	Adult exposure to 25 mg/kg/day DEHP for 2 spermatogenesis cycles	4	123-130	[18]	DMRs induced by DEHP	GEO accession number: GSE174093
Rat	Age	6	65 vs 120	[7]	VMRs in young and mature rats; age-dependent DMRs	[46]
Rat	Perinatal exposure to 0.2 mg/kg BW BDE-47 from gestation day 8 till postpartum day 21	6	65	[7]	DMRs induced by BDE-47	[46]

Table S2. Repetitive elements' counts in all DNA regions and VMRs in mouse sperm

	Count in All (48,948) DNA regions	Count in VMRs (2,566)	% in ALL	% in VMR
DNA	1	0	0.00	0.00
DNA/hAT	1	1	0.00	0.04
DNA/hAT-Ac	3	2	0.01	0.08
DNA/hAT-Charlie	19	0	0.04	0.00
DNA/hAT-Tip100	2	1	0.00	0.04
DNA/TcMar-Tigge	5	0	0.01	0.00
LINE/CR1	20	2	0.04	0.08
LINE/L1	54	9	0.11	0.35
LINE/L1-Tx1	2	0	0.00	0.00
LINE/L2	30	3	0.06	0.12
LINE/Penelope	1	0	0.00	0.00
LINE/RTE-BovB	5	0	0.01	0.00
ALL LINE	112	14	0.23	0.55
Low_complexity	562	8	1.15	0.31
LTR	2	0	0.00	0.00
LTR/ERVL	9	0	0.02	0.00
LTR/ERVL-MaLR	9	0	0.02	0.00
LTR/Gypsy	1	0	0.00	0.00
rRNA	40	19	0.08	0.74
Simple_repeat	3003	59	6.14	2.30
SINE/5S-Deu-L2	2	1	0.00	0.04
SINE/MIR	84	6	0.17	0.23
ALLSINE	86	7	0.18	0.27
srpRNA	55	5	0.11	0.19
tRNA	15	0	0.03	0.00

Table S3. Repetitive elements' counts in all DNA regions and VMRs in rat sperm

Repetitive element	Count in All (279,439) DNA regions	Count in VMRs (9,872)	% in ALL	% in VMR
am RC/Helitron	1	0	0.00	0.00
ARTEFACT	1	0	0.00	0.00
DNA	18	0	0.01	0.00
DNA/Crypton-A	2	0	0.00	0.00
DNA/hAT	1	0	0.00	0.00
DNA/hAT-Ac	2	0	0.00	0.00
DNA/hAT-Blackjack	9	1	0.00	0.01
DNA/hAT-Charlie	290	12	0.10	0.12
DNA/hAT-Tag1	2	0	0.00	0.00
DNA/hAT-Tip100	36	1	0.01	0.01
DNA/MULE-MuDR	1	0	0.00	0.00
DNA/PIF-Harbinger	3	1	0.00	0.01
DNA/PiggyBac	1	0	0.00	0.00
DNA/TcMar-Mariner	4	0	0.00	0.00
DNA/TcMar-Tc2	3	0	0.00	0.00
DNA/TcMar-Tigger	46	3	0.02	0.03
e DNA/TcMar-Tigger	8	0	0.00	0.00
ent DNA/hAT-Charlie	8	1	0.00	0.01
GA tRNA	1	0	0.00	0.00
LINE/CR1	19	1	0.01	0.01
LINE/L1	3312	280	1.19	2.84
LINE/L1-Tx1	2	0	0.00	0.00
LINE/L2	55	1	0.02	0.01
LINE/RTE-BovB	3	0	0.00	0.00
LINE/RTE-X	3	0	0.00	0.00
ALL LINE	3394	282	1.21	2.86
Low_complexity	1391	44	0.50	0.45
LTR	6	0	0.00	0.00
LTR/ERV1	408	23	0.15	0.23
LTR/ERVK	2864	210	1.02	2.13
LTR/ERVK	1	0	0.00	0.00
LTR/ERVL	689	26	0.25	0.26
LTR/ERVL-MaLR	3168	184	1.13	1.86
LTR/Gypsy	7	0	0.00	0.00
t LTR/ERVK	13	2	0.00	0.02
ALL LTR	7156	445	2.56	4.51
Retroposon/L1-dep	370	27	0.13	0.27

rRNA	77	6	0.03	0.06
Satellite	53	2	0.02	0.02
scRNA	19	2	0.01	0.02
Simple_repeat	7830	256	2.80	2.59
SINE/5S-Deu-L2	2	0	0.00	0.00
SINE/Alu	2300	73	0.82	0.74
SINE/B2	2286	110	0.82	1.11
SINE/B4	2319	59	0.83	0.60
SINE/ID	1219	66	0.44	0.67
SINE/MIR	131	2	0.05	0.02
SINE/tRNA-RTE	1	0	0.00	0.00
ALL SINE	8258	310	2.96	3.14
snRNA	21	0	0.01	0.00
srpRNA	10	1	0.00	0.01
tRNA	37	0	0.01	0.00
Unknown	124	1	0.04	0.01
v tRNA	20	0	0.01	0.00

Table S4. Overlap between DNA regions associated with histones in spermatogenic cells and age-dependent DMRs in mouse sperm. Numbers in bold indicate significant difference (Fisher's p <0.05) between DMRs and non-DMRs and between hypo- and hypermethylated DMRs.

Cell type for open regions	% overlapping with histone regions			
	DMRs	non-DMRs	hypomethylated DMRs	hypermethylated DMRs
**All histones***				
Spermatogonia	**79.4**	**83.9**	**75.9**	**81.1**
Spermatocytes	**85.0**	**87.3**	**82.7**	**86.1**
Spermatids	**75.9**	**80.7**	**71.8**	**77.9**
**H3K27ac**				
Spermatogonia	**50.9**	**60.0**	**47.5**	**52.6**
Spermatocytes	**29.1**	**43.0**	**18.1**	**34.5**
Spermatids	**23.4**	**33.8**	**21.0**	**24.6**
**H3K27me3**				
Spermatogonia	**19.9**	**17.3**	**17.7**	**21.0**
Spermatocytes	**58.8**	**70.1**	**48.5**	**63.8**
Spermatids	43.0	43.0	**39.6**	**44.6**
**H3K4me1**				
Spermatogonia	**6.5**	**4.8**	**5.6**	**6.9**
Spermatocytes	**47.0**	**51.0**	**42.9**	**48.9**
Spermatids	44.1	44.9	**41.5**	**45.4**
**H3K4me2**				
Spermatogonia	37.9	37.4	**35.0**	**39.2**
Spermatocytes	**44.1**	**50.4**	**38.5**	**46.9**
Spermatids	**37.1**	**43.0**	**34.5**	**38.4**
**H3K4me3**				
Spermatogonia	**68.0**	**75.6**	**63.8**	**70.1**
Spermatocytes	**81.6**	**85.1**	**78.4**	**83.1**
Spermatids	**58.1**	**69.4**	**54.6**	**59.8**
**H3K9me3**				
Spermatogonia	**1.21**	**0.76**	1.14	1.25
Spermatocytes	**1.09**	**0.55**	**2.14**	**0.59**
Spermatids	**0.69**	**0.31**	0.61	0.72

Table S5. DNA motifs enriched in mouse sperm VMRs.

#	Motif	*P*-value	*E*-value	% non-VMRs with motif	% VMRs with motif
1	AARATGGMWW	4.9e-011	8.4e-010	70.1	90.5
2	CCGGTGAS	3.7e-007	6.2e-006	2.0	5.1
3	AGTGTATCCTGCACA	5.9e-005	1.0e-003	0.03	1.2
4	AGGGTCACAAGTCC	1.3e-004	2.3e-003	0.2	1.2
5	AAGACCCCGAACCG	2.1e-004	3.6e-003	0.002	0.6
6	ATACAGAC	4.6e-003	7.8e-002	1.3	3.6
7	GGTTCCGGGACYCC	6.3e-003	1.1e-001	0.6	3.5
8	CAGGCAAAGCSCTCC	1.0e-002	1.7e-001	0.3	2.4
9	AGGACAGGTGTCCGC	2.3e-002	4.0e-001	0.4	1.9
10	AGCATCAGM	3.0e-002	5.1e-001	4.1	8.2
11	AGCACCGGGGTASC	3.1e-002	5.2e-001	0.1	1.0
12	AGCAGAWA	3.1e-002	5.2e-001	37.4	49.9
13	CCTCCCAAG	3.8e-002	6.5e-001	0.2	0.9
14	GACTCTGCGCCC	4.5e-002	7.7e-001	0.1	0.8
